# The C/EBP Homologous Protein (CHOP) Transcription Factor Functions in Endoplasmic Reticulum Stress-Induced Apoptosis and Microbial Infection

**DOI:** 10.3389/fimmu.2018.03083

**Published:** 2019-01-04

**Authors:** Hai Hu, Mingxing Tian, Chan Ding, Shengqing Yu

**Affiliations:** Department of Veterinary Public Health, Shanghai Veterinary Research Institute, Chinese Academy of Agricultural Sciences, Shanghai, China

**Keywords:** C/EBP homologous protein, endoplasmic reticulum stress, apoptosis, microorganisms, virus, bacteria

## Abstract

Apoptosis is a form of cell death by which the body maintains the homeostasis of the internal environment. Apoptosis is an initiative cell death process that is controlled by genes and is mainly divided into endogenous pathways (mitochondrial pathway), exogenous pathways (death receptor pathway), and apoptotic pathways induced by endoplasmic reticulum (ER) stress. The homeostasis imbalance in ER results in ER stress. Under specific conditions, ER stress can be beneficial to the body; however, if ER protein homeostasis is not restored, the prolonged activation of the unfolded protein response may initiate apoptotic cell death via the up-regulation of the C/EBP homologous protein (CHOP). CHOP plays an important role in ER stress-induced apoptosis and this review focuses on its multifunctional roles in that process, as well as its role in apoptosis during microbial infection. We summarize the upstream and downstream pathways of CHOP in ER stress induced apoptosis. We also focus on the newest discoveries in the functions of CHOP-induced apoptosis during microbial infection, including DNA and RNA viruses and some species of bacteria. Understanding how CHOP functions during microbial infection will assist with the development of antimicrobial therapies.

## Introduction

The endoplasmic reticulum (ER) is an important organelle in eukaryotic cells and plays important roles in protein synthesis, modification and processing, folding, assembly, and the transportation of nascent peptide chains (1, 2). The ER has a strong homeostasis system and the stability of the internal environment is the basis for the ER to achieve its functions (3). Some physiological and pathological conditions, including changes in temperature and pH, the accumulation of damaged DNA, contamination with toxic effluents, and infection with viruses and bacteria can cause ER stress (4). ER stress can be divided into three types, including the unfolded protein response (UPR), the ER overload response, and sterol regulatory elements combined with protein-mediated regulatory responses (5). ER stress usually refers to the UPR, which occurs when the misfolded or unfolded proteins in the ER increase and activate the stress signal that transmits to the nucleus through the ER membrane. Upon ER stress, cells mainly elicit two responses: one leading to cellular survival and the other leading to apoptosis (6). Using the survival pathway, cells conquer such disadvantageous effects and maintain homeostasis through the UPR, inhibiting the transcription of mRNA, enhancing the folding capacity of the ER, and ERAD (ER-assisted degradation) to restore homeostasis (7). Under chronic or overwhelming ER stress, the normal functions of the ER fail to recover, resulting in cellular dysfunction and apoptosis (8).

Many ER stress related diseases have been reported in clinical populations (9, 10). When ER stress occurs with high intensity, or it is prolonged, homeostasis is not restored and apoptosis is induced by ER-related molecules. ER-induced apoptosis occurs via three primary pathways, including the IRE1/ASK1/JNK pathway, the caspase-12 kinase pathway, and the C/EBP homologous protein (CHOP)/GADD153 pathway (11, 12). The IRE1/ASK1/JNK pathway is important for apoptosis in the ER and has been found active during many diseases, such as osteoporosis, urothelial carcinoma (13, 14). The caspase-12 kinase pathway is also involved in many diseases, neonatal hypoxic-ischemic encephalopathy, parkinson's disease, etc. (15–17). The CHOP pathway plays an important role in ER stress-induced apoptosis due to pathogenic microbial infections, neurological diseases and neoplastic diseases.

## The Structure and Characterization of CHOP

The CHOP protein was first identified during methyl methanesulfonate research involving UV irradiation and alkylation (18). CHOP belongs to the family of CCAAT/enhancer binding proteins (C/EBPs) and is involved in the regulation of genes that encode proteins involved in proliferation, differentiation and expression, and energy metabolism. CHOP is a 29 kD protein with 169 (human) or 168 (rodents) amino acid residues. It contains two functional domains, including an N-terminal transcriptional activation domain and a C-terminal basic-leucine zipper (bZIP) domain (19). Deletion mutant analyses showed that the bZIP domain plays a crucial role in CHOP-induced apoptosis (20). Research showed that CHOP-deficient cells were resistant to ER stress-induced apoptosis (11, 21). CHOP deficient mouse experiments revealed that CHOP-induced apoptosis is relevant to many diseases that cause ER stress (22, 23). Apoptosis caused by the CHOP pathway is becoming the focus of an increasing number of researchers (24–26).

The collective findings of CHOP research have indicated that this factor plays an important role in ER stress-mediated apoptosis. Here, we summarize the recent findings on the functions of CHOP during ER stress-induced apoptosis and microbial infection.

## The Functions of CHOP in ER Stress-induced Apoptosis

### Upstream Regulatory Pathway of CHOP

There are a series of precise mechanisms in cells, which ensure the correct folding and assembly of intracellular proteins. Thus, only correctly folded proteins can be transported out of the ER to perform their functions. During normal physiology, CHOP is ubiquitously expressed at very low levels (27). However, pathological conditions or microbial infection caused ER stress is overwhelming, the expression of CHOP rises sharply and apoptosis is activated, and this process can occur in a wide variety of cells (27, 28). Those processes are mainly regulated by three factors, including protein kinase RNA-like endoplasmic reticulum kinase (PERK), activating transcription factor 6 (ATF6), and inositol requiring protein 1 (IRE1) (11).

#### PERK

PERK is a transmembrane protein and an important sensor that participates in the UPR by attenuating protein translation and regulating oxidative stress (29). Unfolded proteins in the ER stimulate PERK oligomerization and autophosphorylation, and can phosphorylate eukaryotic translation initiation factor 2α (eIF2α) (29). Phosphorylation of eIF2α promotes the transcription of ATF4, which converges on the promoters of target genes, including CHOP, GADD34, and ATF3 (1, 30, 31). Research shows that PERK^−/−^and ATF4^−/−^cells and eIF2α (Ser51Ala) knock-in cells fail to induce CHOP during ER stress (32). The PERK/ATF4/CHOP signaling pathway is considered to play a pivotal function in inducing cell apoptosis, both *in vitro* and *in vivo* (33–35). However, research shows that CHOP may not fully induce cell death, and that CHOP and ATF4 cooperation is required for the induction of cell death (31).

#### ATF6

ATF6 is a transmembrane protein. Under ER stress, ATF6 translocates to the Golgi compartment where it is cleaved and activated (36). When ATF6 is activated, it translocates to the nucleus as a homo-or heterodimer and interacts with ATF/cAMP response elements and ER stress-response elements (1). Such complexes bind the promoters of several genes involved in UPR (such as CHOP, GRP78, XBP1) and induce target-gene transcription (37, 38). Along with XBP1(s), ATF6 contributes to the augmentation of ER size and ER protein-folding capacity through target genes. ATF6 can activate the transcription of both CHOP and XBP-1, while XBP-1 can also regulate the expression of CHOP. Thus, ATF6 can cooperate with XBP-1 to activate CHOP (39).

#### IRE1

**Figure 1:**
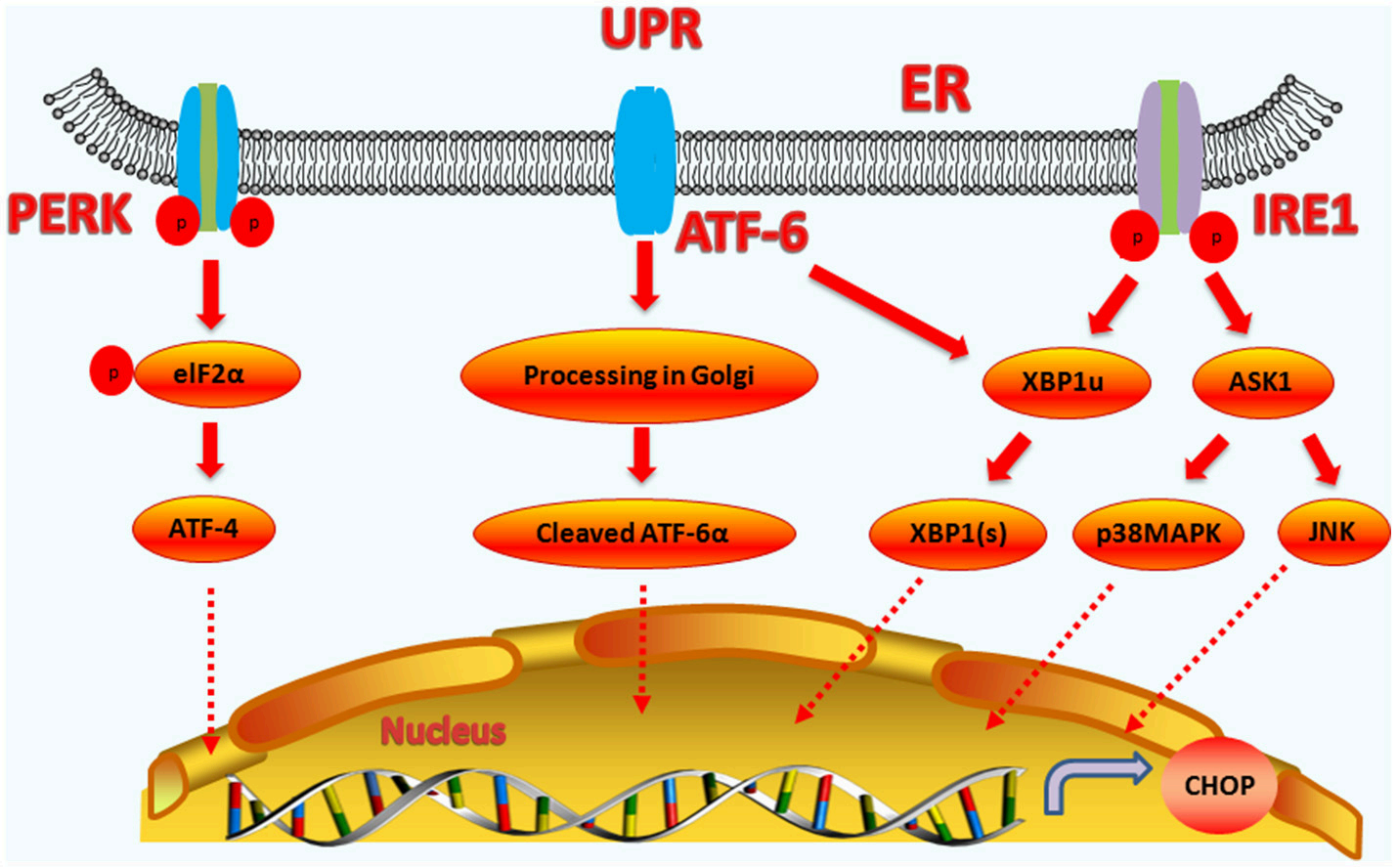
Upstream regulatory pathway of CHOP. The three signaling branches of UPR lead to CHOP transcription. Activated PERK phosphorylates eIF2α, which results in the translation of ATF4. Subsequently, ATF4 translocates to the nucleus, which increases the expression of CHOP and ATF3. CHOP and ATF3 then bind to the promoters downstream genes. ATF6 translocates to Golgi apparatus, where it is activated by proteolysis. Activated ATF6 transcriptionally upregulates CHOP expression. Additionally, ATF6 can regulate XBP-1 to activate CHOP. On one hand, the activation of IRE1α processes unspliced XBP1 mRNA to create activated XBP1(s), which enters the nucleus and controls the expression of CHOP. On the other hand, IRE1α activates apoptotic signaling kinase 1 (ASK1), which in turn phosphorylates p38MAPK and JNK to activate CHOP.

IRE1 is a transmembrane protein containing two functional domains, including an N-terminal luminal sensor domain and a C-terminal cytosolic effector (40). IRE1 contains protein kinase and endoribonuclease activities. Unfolded proteins in the ER stimulate IRE1α oligomerization and autophosphorylation, which activates the endoribonuclease activity (41). Upon activation, IRE1α splices the substrate precursor, XBP-1, mRNA introns to produce a mature and active XBP-1 protein (42, 43). The active protein then binds the promoters of several genes involved in UPR and ERAD, and regulates gene expression (such as CHOP) to restore protein homeostasis. Thus, CHOP expression can be upregulated by XBP1(s) (44–46). IRE1α can stimulate the activation of the apoptotic-signaling kinase-1 (ASK1), which then activates the downstream kinases, Jun-N-terminal kinase (JNK) and p38 mitogen-activated protein kinase (p38 MAPK), which cause apoptosis (40). The P38 MAP kinase family phosphorylates Ser78 and Ser81 of CHOP, which induces cell apoptosis (47, 48). Moreover, during tunicamycin-induced apoptosis, the JNK inhibitor, SP600125, could suppress CHOP upregulation and subsequent death receptor 5 (DR5) expression, indicating that JNK activation is also involved in the modulation of CHOP (49). JNK and p38 MAPK can also promote the phosphorylation and activation of the pro-apoptotic protein, BAX, to regulate cell apoptosis (50). Therefore, CHOP can cooperate with JNK and p38 MAPK to regulate cell apoptosis (the upstream regulatory pathways of CHOP are summarized in Figure 1).

### Downstream Regulatory Pathway of CHOP

#### CHOP Induces Apoptosis Through Mitochondria-Dependent Pathway

A variety of upstream pro-apoptotic signals act on the mitochondrial membrane. Active BCL2-family proteins form protein channels in the mitochondrial membrane and the opening of the mitochondrial PT hole enables the apoptotic active substances (such as cytochrome c, Smac, etc.) to be released into the cytoplasm (51). Such events cause downstream caspase-family proteins to activate and act on the corresponding substrates leading to apoptosis.

As a transcription factor, CHOP can regulate the expression of many anti-apoptotic and pro-apoptotic genes, including genes encoding the BCL2-family proteins, GADD34, TRB-3, and DOCs (52, 53). In the CHOP-induced apoptotic pathway, CHOP regulates the BCL2 protein family. The BCL2 family consists of 25 members that share up to four conserved motifs known as BCL2 homology domains (BH1–4) (54). The BCL2 protein family can be divided into two categories: anti-apoptotic proteins and proapoptotic proteins (55). Anti-apoptotic proteins mainly include BCL2, BCL-XL, MCL-1, and BCL-W, while the proapoptotic proteins can be divided into two categories: multidomain and BH3-only domain proteins (56, 57). Multidomain proteins include BAK, BAX, and BOK, while the BH3-only domain proteins include BID, BIM, BAD, BIK, NOXA, and PUMA (58, 59). The BH3-only proteins regulate cell apoptosis mainly by inhibiting the expression of the BCL2 anti-apoptotic protein, or promoting the expression of multidomain proteins, such as BAX (60).

Under ER stress, CHOP can function as either a transcriptional activator or repressor. It forms heterodimers with other C/EBP family transcription factors via bZIP-domain interactions to inhibit the expression of genes responsive to C/EBP family transcription factors, while enhancing the expression of other genes containing a specific 12–14 bp cis-acting element (19). CHOP can downregulate the expressions of BCL2, BCL-XL, and MCL-1, and upregulate the expression of BIM, causing increased BAK and BAX expression (60, 61). After BAX-BAK oligomerization, the oligomers cause the release of apoptotic factors such as cytochrome c (Cyt-C) and apoptosis-inducing factor (AIF) through mitochondria permeabilization, eventually cause cell death (62).

**Figure 2:**
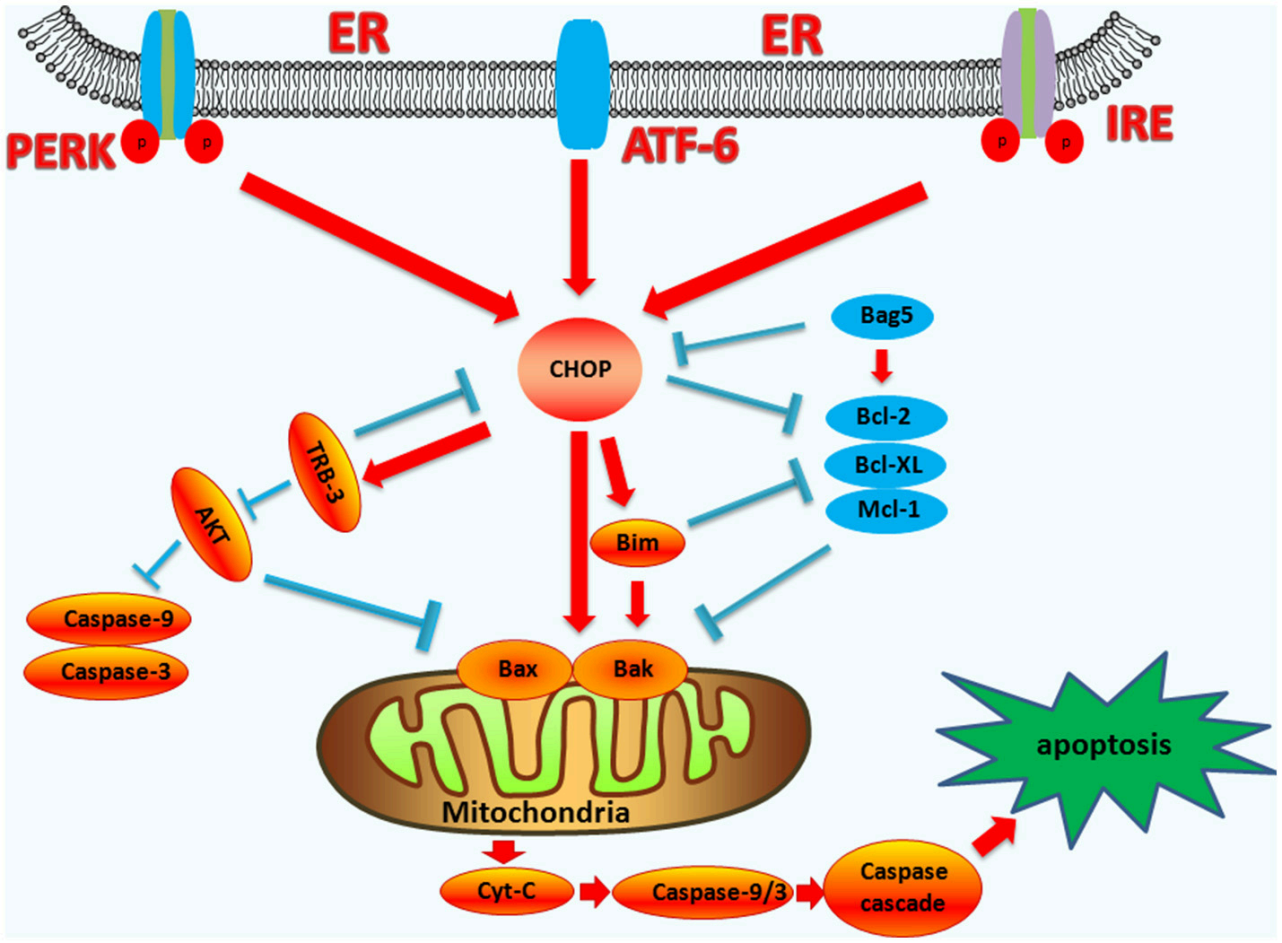
The functions of CHOP in endogenous-pathway-induced apoptosis. CHOP triggers the intrinsic apoptotic pathway through the inhibition of BCL-2, BCL-XL, MCL-1, and the upregulation of BIM, which regulates BAX-BAK-mediated mitochondrial outer membrane permeabilization. This leads to cytochrome c release and the caspase cascade. Bag5 can reduce CHOP expression and increase Bcl-2 gene expression. CHOP can also regulate the apoptosis of cells by up-regulating the expression of the TRB3 gene, preventing Akt phosphorylation, which inhibits the activity of caspases-3/9.

TRB3 is an intracellular pseudokinase that modulates the activity of the signal transduction cascade and is highly regulated in many cells (63, 64). Research has shown that under conditions of hypoxia and ER stress in non-cardiac cells, TRB3 increases its expression (65). During ER stress, TRB3 is upregulated by the ER stress-inducible transcriptional factor, ATF4-CHOP (66). CHOP interacts with TRB3, which contributes to the induction of apoptosis (67, 68). The binding site of CHOP overlaps the amino acid response elements in the TRB3 promoter, and the specific regions in CHOP and TRB3 are responsible for their interaction (69). The expression of TRB3 can inhibit AKT activity and has a pro-apoptotic capacity (70, 71). AKT directly modulates the expression of caspase-3 and caspase-9, as well as the mitochondrial pro-apoptotic proteins, BAX, and BAD (72). TRB3 can also inhibit the anti-apoptotic activity of AKT by inhibiting the phosphorylation of the Ser473 and Thr308 sites of AKT (63, 73). The upregulation of TRB3 expression can be accompanied by the activation of caspase-3, thereby enhancing apoptosis. Interfering with the expression of TRB3 partially attenuates caspase-3 activity (74, 75), and therefore, CHOP also regulates apoptosis by up-regulating the expression of the TRB3 gene, and directly or indirectly affecting the activity of caspase (the endogenous pathway-induced apoptosis pathways are summarized in Figure 2).

#### CHOP Induces Apoptosis Through Death-Receptor Pathway

Cell death induced by ER stress also can be mediated through exogenous pathways. Death receptor-mediated apoptosis occurs via death ligands (Fas, TNF, and TRAIL) combined with death receptors. The receptor protein, Fas-associated death domain protein, is recruited to form a death-inducing signal complex, which activates the cytosolic caspase-8, which then activates the downstream caspase to induce apoptosis.

The PERK-ATF4-CHOP pathway can induce apoptosis by binding to the death receptor pathway and upregulating the expression of death receptor 4 (DR4) and DR5. CHOP regulates DR4 or DR5, or both DR4 and DR5 to induce apoptosis is dependent on the different cell types and stimulus. The TRAIL-R1/DR4 death receptors can be activated by ER stress. CHOP interacts with the phosphorylated transcription factor JUN to form a complex that binds to the promoter region of DR4 in lung cancer cells (76). In giant brain neuronal cells (GCN5), the N-terminal domain of CHOP interacts with phosphorylated JUN to form a complex that regulates the expression of DR4 and DR5 (76). CHOP also upregulates the expression of DR5 by binding to the 5′-region of the DR5 gene (77). ATF3 is also involved in mediating DR5 production. In a colon cancer cell model in which the p53 gene is deleted, the ATF3 gene involved in ER is also involved in mediating DR5 production (78).

**Figure 3:**
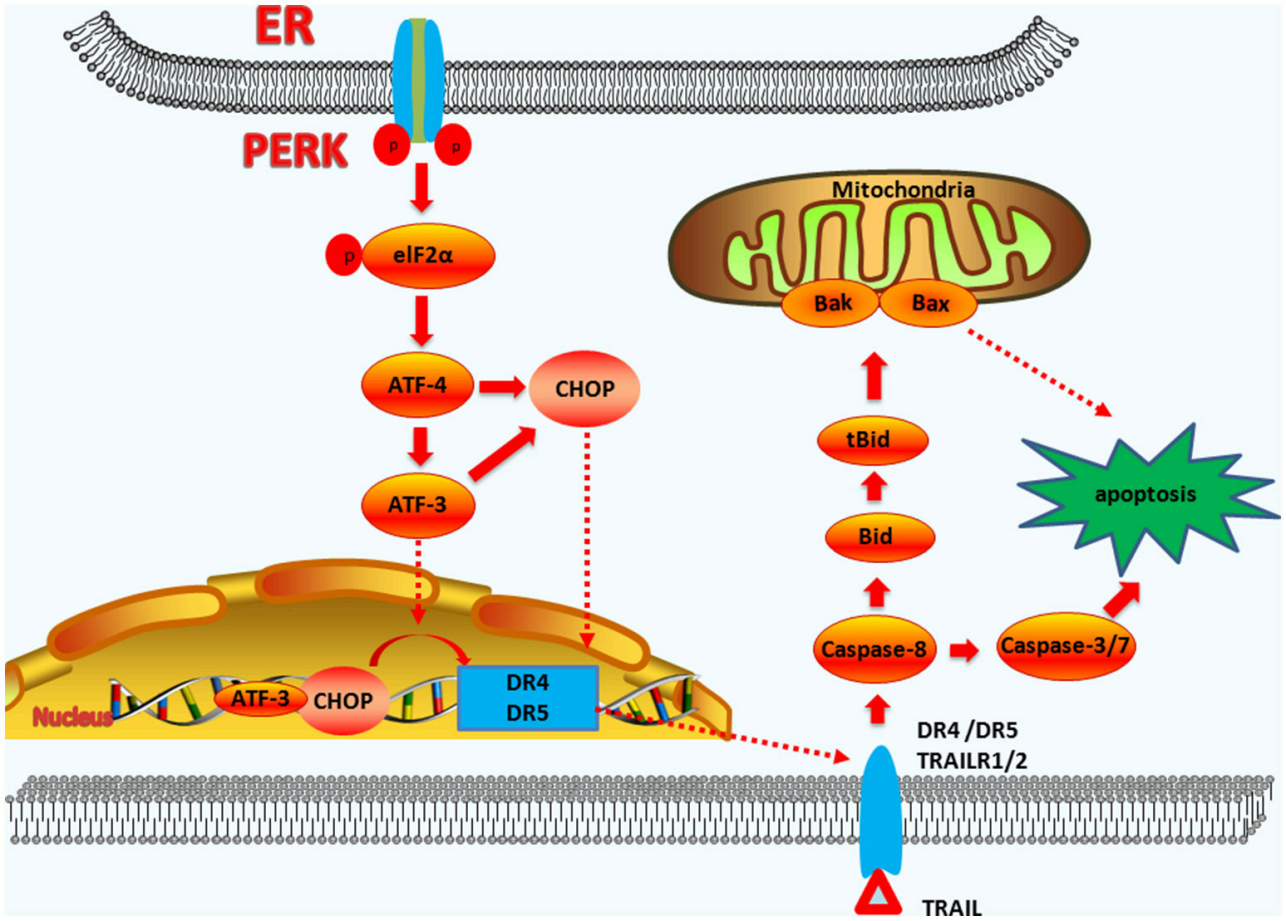
The functions of CHOP in exogenous-pathway-induced apoptosis. CHOP triggers the extrinsic apoptotic pathway through the upregulation of DR4 and DR5. PERK can induce ATF4 expression, which results in CHOP and ATF3 expression. CHOP and ATF3 then bind to the promoters of the DR4 and DR5 genes, upregulating their expression. TRAIL combining with DR4 and DR5 regulates the caspase 8-mediated cascade, which activates caspase3/7 to lead apoptosis. On the other hand, Bid is cleavaged into tBid. tBid then regulates the BAX-BAK-mediated mitochondrial apoptosis pathways.

Research shows that the CHOP-DR5 signaling sensitizes several chemically challenged cancer cells to extrinsic apoptosis mediated by reactive oxygen species (ROS), *in vitro* (79, 80). If the ER stress is not reversible, PERK-CHOP function will persist, permitting DR5 mRNA to rise. The accumulation of DR5 in the ER and Golgi can drive ligand-independent multimerization of the long splice variants of death receptor 5 (DR5L). DR5L accelerates the formation of the death-inducing signaling complex (DISC) and activates caspase-8 (81). The activation of caspase-8 can also cleave BID located in the cytoplasm into tBID. tBID has powerful pro-apoptotic activity and can act on the mitochondrial membranes with BAK and BAX, which cause Cyt-C release. Subsequently, this leads to apoptosis via the exogenous and the endogenous pathways (82) (the exogenous pathway-induced apoptosis pathways are summarized in Figure 3).

#### CHOP Induces Apoptosis Through Other Pathways

In addition to mediating apoptosis through the endogenous and exogenous pathways, CHOP also mediates apoptosis through other pathways. CHOP can increase the expression of the ERO1α (ER reductase) gene, which catalyzes the oxidation of protein disulfide isomerase (PDI), resulting in the production of H_2_O_2_ in the ER. The highly oxidized state of the ER (22, 83) results in H_2_O_2_ leakage into the cytoplasm, induces the production of ROS and a series of apoptotic and inflammatory reactions (84–86).

High ROS concentrations in the lumen of the ER activate the IP3R1 calcium ion release channel, allowing calcium ions to enter the cytosol. Cytoplasmic calcium promotes ROS by activating the calcium-sensing kinase, CaMKII (calcium-dependent protein kinase), and NOX2, a subunit of NADPH oxidase on the cell membrane. Those factors then promote the transcription of CHOP, resulting in apoptosis (87). This is a positive feedback pathway. The CHOP-CaMKII pathway also activates JNK, which is involved in ER stress-induced apoptosis by inducing the expression of Fas, NOX2, and oxidative stress (88, 89). In some models of diabetes, the deletion of CHOP can inhibit the apoptosis of pancreatic beta cells, and provides a protective mechanism to reduce the production of ERO1α, which subsequently reduces markers of oxidative stress and the expression of antioxidant genes (90). Moreover, ROS scavengers can attenuate the expression of PERK/eIF2α/CHOP pathway-related proteins (91).

The CHOP gene also directly activates GADD34 (DNA damage protein), which combines with phosphatase 1 protein (PP1) and dephosphorylates eIF2α, resulting in protein translation recovery and increases of ER stress and cell apoptosis (92, 93). Under ER stress, cells with GADD34 mutations can significantly reduce protein complex formation compared to normal cells.

The overexpression of CHOP can lead to cell cycle arrest and result in cell apoptosis. At the same time, CHOP-induced apoptosis can also trigger cell death by inhibiting the expression of cell cycle regulatory protein, p21. The p21 protein not only inhibits the G1 phase of the cell cycle, but also has a close relationship with the activity of pre-apoptotic factors. Thus, the relationship between CHOP and p21 may explain changes in the state of the cell from adapting to ER stress to pre-apoptotic activity (94, 95).

Under most conditions, CHOP can directly bind to the promoters of downstream related genes, while under specific condition, CHOP can cooperate with other transcription factors to affect apoptosis. Recent studies have shown that Bcl-2-associated athanogene 5 (Bag5) is over-expressed in prostate cancer and inhibits ER stress-induced apoptosis. Overexpression of Bag5 results in decreased CHOP and BAX expression, and increased Bcl-2 gene expression (96). Bag5 overexpression inhibited ER stress-induced apoptosis in the UPR by suppressing PERK-eIF2-ATF4 and enhancing the IRE1-Xbp1 activity (97).

CHOP has also been reported to regulate the expression of BH3-only proteins by interacting with FOXO3A (in neuronal cells treated with tunicamycin) and the AP-1 complex protein, cJUN, leading to its phosphorylation. Knockdown of CHOP prevented the dephosphorylation of its downstream target FOXO3a (Thr32) (58).

Synoptically, CHOP-dependent apoptosis is mainly mediated by directly or indirectly altering the expression of pro-apoptotic or anti-apoptotic genes. Collectively, the apoptotic pathways are mediated by the downstream targets of CHOP; however, the molecular interaction mechanisms by which this occurs remain to be understood.

### CHOP Functions During Apoptosis and Autophagy

Autophagy is a lysosomal degradation system involving the degradation and recycling of obsolete, damaged, or harmful cytoplasmic materials, and organelles. Autophagy generally precedes apoptosis under cellular stresses and the apoptotic program is activated when such stresses exceed a critical duration or intensity threshold (98). Here, we describe the important roles of CHOP in ER stress induced apoptosis. Recent studies have identified CHOP as a direct regulator of numerous genes involved in the pro-survival autophagic process (99, 100). CHOP has been implicated in autophagy induced by amino acid starvation, ER stress, and infection with microorganisms (101, 102). Research has shown that knockdown of CHOP not only enhances tunicamycin-induced autophagy, but also significantly attenuates ER stress-induced apoptosis in human colon cancer cells. Moreover, CHOP modulates the induction of autophagosomes during ER stress, as evidenced by the inhibition of LC3-II expression and GFP-LC3B dots (103). Another study also showed that UPR-activated CHOP elicited complete autolysosome maturation during hepatitis C virus-induced autophagy via an LC3B-II-dependent mechanism (104). During amino acid starvation and ER stress, CHOP can up-regulate the expression of autophagy-related genes at the onset, while in the later stages of starvation, CHOP can inhibit the occurrence of autophagy and gradually initiate apoptosis (105). Therefore, CHOP plays a pivotal role in switching between apoptosis and autophagy.

## The Functions of CHOP-induced Apoptosis During Microbial Infection

Many pathogenic microorganisms infect host cells and can cause ER stress (106). When ER stress occurs at a high intensity or is prolonged, homeostasis is not restored and apoptosis will occur. Many microbes infect host cells to induce apoptosis through regulating the expression of CHOP. CHOP-induced apoptosis can be beneficial to the microorganisms or be detrimental to their growth and reproduction (Table 1). Here, we summarize recent findings in the functions of CHOP-induced apoptosis during microbial infection.

**Table 1:** The functions of CHOP in ER stress-induced apoptosis in microbial models.

**Microbial type**	**Key proteins**	**Involved mechanisms and phenotype**	**Functions of CHOP**	**Research models**	**References**
Porcine circovirus type 2	CHOP, ATF4 Caspase 3,	PERK-eIF2α-ATF4 -CHOP-BCL2	Apoptosis and beneficial to PCV2 replication	PK-15 cells	(107, 108)
Infectious bronchitis virus	CHOP, TRIB3, BCL2	PERK-eIF2α-ATF4 PKR-eIF2α-ATF4	Apoptosis and facilitates virus release	H1299 cells	(109, 110)
Newcastle disease virus	CHOP, ATF4, cyclin D1	PERK-ATF4-CHOP	Apoptosis and beneficial to NDV replication	Asynchronously growing cells	(111)
West Nile virus	CHOP, PARP, Caspase 3,	PERK-ATF4-CHOP	Apoptosis and limit WNV replication	Human neuroblastoma cells,	(112)
Coxsackie virus B3	CHOP, BAX, Caspase 3,	CHOP-BAX- Caspase 3	Apoptosis and beneficial to CVB3 replication	Mice	(113)
Japanese encephalitis virus	CHOP, p38, BCL2	p38-CHOP-BCL2	Apoptosis	BHK-21 cells	(114, 115)
HIV	CHOP, BCL2, BAX	XBP-1-CHOP- Caspase 3/9	Apoptosis	Human brain endothelial cells	(116, 117)
*M. tuberculosis*	CHOP, Caspase3/9	p-eIF2α-CHOP	limits *M. tuberculosis* replication	RAW 264.7 cells	(26, 118)
*Helicobacter pylori*	CHOP, BIM, BAX, TRIB3	PERK-CHOP PKR-eIF2α-ATF4	Damage the cell and tissue (gastric injury)	AZ-521 cells	(119, 120)
*Escherichia coli*	CHOP, DR5, TRAIL	CHOP-DR5- Caspase 3/8	Damage the cell and tissue (renal failure)	THP-1 cells	(121, 122)
*Shigella dysenteriae*	CHOP, DR5	p38-CHOP-DR5	Damage the cell and tissue (neurological abnormalities)	RPE cells	(123)

### The Functions of CHOP in Viral Infection

After viruses invade the host they must adapt to the intracellular environment, and use host cell factors to self-replicate. The ER is an essential organelle for virus assembly and replication, and the replication and proliferation of the virus can affect the function of the ER and cause ER stress. Under prolonged ER stress, cell apoptosis is activated. Under certain conditions, apoptosis is beneficial or essential for virus replication and the release of virus particles. Under other conditions, apoptosis can inhibit the proliferation and spread of the virus in host cells to protect uninfected cells.

After DNA viruses infect host cells, CHOP plays a critical role in apoptosis induced by ER stress. Porcine circovirus type 2 (PCV2) has been reported to elicit the UPR and mediate apoptosis following ER stress (107). PCV2 triggers the UPR in PK-15 cells by activating the PERK-eIF2α-ATF4-CHOP pathway, without the concomitant activation of IRE1 or ATF6. Over-expression of GRP78 can enhance viral capsid expression and/or viral titers. Thus, PCV2 deploys the UPR to enhance its replication (107). Replicase (Rep) and capsid (Cap) proteins of PCV2 activate the eIF2α-ATF4-CHOP pathway. Cap expression significantly reduced the expression of anti-apoptotic BCL2 and increased caspase-3 cleavage by increasing the expression of CHOP. Knockdown of PERK by RNA interference significantly reduces Cap-induced CHOP expression and caspase-3 cleavage. Cap induces UPR and apoptosis via the PERK-eIF2α-ATF4-CHOP-BCL2 pathway (108). When PERK is inhibited by GSK2606414 or eIF2α dephosphorylation is suppressed by salubrinal, viral replication is limited, suggesting that CHOP is involved in apoptosis induced by PCV2 and may be beneficial to viral replication (107).

CHOP also plays an important role in RNA virus-infected host cells. H1299 cells infected with Infectious Bronchitis Virus (IBV) induced apoptosis via ER stress. Post-IBV infection, the IRE1α-XBP1 pathway of the UPR was activated. IRE1α protects infected cells from IBV-induced apoptosis, which requires both its kinase and RNase activities. IRE1α antagonizes IBV-induced apoptosis by modulating the phosphorylation of the proapoptotic c-Jun N-terminal kinase (JNK) and the pro-survival RAC-alpha serine/threonine-protein kinase (Akt) (124). At the same time, IBV infection also activates two other pathways, including PERK-eIF2α-ATF4 and PKR-eIF2α-ATF4. Following activation, ATF4, ATF3 and CHOP are upregulated. CHOP affects GADD34 and the dephosphorylation of eIF2α. This leads to the recovery of protein translation and increasing ER stress and cell apoptosis. CHOP can up-regulate the expression of the apoptotic precursor protein, pseudokinase tribbles homolog 3 (TRIB3), which inhibits the ERK pro-survival pathway, thereby promoting apoptosis. During apoptosis induced by IBV, CHOP also decreases the expression of BCL2, which contributes to CHOP-mediated apoptosis. Thus, IBV induces apoptosis through the IRE/JNK and PERK/PKR pathways. More importantly, in CHOP-deficient cells, IBV-induced apoptosis is attenuated and virus replication is inhibited. Thus, all such results suggest that CHOP-induced apoptosis is beneficial to IBV replication (109, 110).

Coxsackie virus B3 (CVB3) can trigger the UPR and induce apoptosis by mediating the production of CHOP (125, 126) and reducing the cardiac Bcl-2/Bax ratio. That finding supports that CHOP-mediated apoptosis plays a role in acute viral myocarditis (AVMC), which occurs primarily through a mitochondria-dependent pathway. However, the precise mechanism leading to CHOP-mediated apoptosis remains unclear and requires further investigation. Researches show that CHOP deficiency reduces CVB3 replication, cardiac damage, and promotes survival in CVB3-mediated acute viral myocarditis, *in vivo* (113, 125).

CHOP-mediated apoptosis in premature cells may function as a host defense response by limiting virus replication and pathogenesis. West Nile Virus (WNV) can induce CHOP expression through the PERK-ATF4-CHOP pathway, and CHOP induces the expression of the downstream target gene, GADD34. eIF2α dephosphorylation also occurs, leading to the restoration of protein translation, which increases ER stress and cell apoptosis. Simultaneously, CHOP induces caspase-3 activation, leading to apoptosis. In CHOP-deficient mouse embryonic fibroblasts (MEFs), WNV grows to significantly higher viral titers than that in wild-type MEFs, suggesting that CHOP-mediated apoptosis functions to control WNV replication *in vitro* (112, 127).

### The Functions of CHOP in Bacterial Infection

ER stress-induced apoptosis plays an important role in bacteria-infected host cells. The prevention of apoptosis provides a survival advantage because it facilitates bacterial replication inside host cells (128).

*Mycobacterium tuberculosis* (*Mtb*) infected host cells activate three signaling pathways of ER stress (IRE1, PERK, and ATF6) (129). Recent evidence suggests that *Mtb* and its 38 kDa antigen can activate the PERK/eIF2α/CHOP pathway (25, 26). *Mycobacterium tuberculosis* and its antigens have been shown to be associated with IRE1α/TRAF2/ASK1/JNK/p38MAPK activation and to result in apoptosis (130). Lim et al. reported that CHOP production induced by the 38 kDa antigen decreased when the JNK pathway was inhibited (25). In A549 cells, JNK phosphorylated Bcl-2 to inhibit its anti-apoptotic activity, and also phosphorylated Bax. Phosphorylated Bax then translocated to the mitochondria and activated Bak to promote apoptosis (130). Thus, CHOP can cooperate with IRE1α/TRAF2/ASK1/JNK to regulate the occurrence of apoptosis.

During *M. tuberculosis* infection, excessive expression of CHOP can promote cell apoptosis by at least two ways: (i) activation of ERO1α. The 38 kDa antigen induces the production of ROS and the subsequent ERS via ERO1α, which leads to apoptosis through high concentrations of peroxide in the ER environment (25). (ii) Inhibition of Bcl-2. Researchers have reported that ESAT-6 induces CHOP to form dimers with CREB, leading to decreased Bcl-2 expression and increased Bax expression (130, 131). More importantly, when CHOP is interfered with siRNA, it can significantly increase the survival of *M. tuberculosis* in host cells (26). Collectively, CHOP-induced apoptosis is beneficial to combat *Mtb* infection.

Vacuolating cytotoxin (VacA) is a critical virulence factor of *Helicobacter pylori*. VacA can upregulate the expression of CHOP after gastric epithelial cells are stimulated with cytotoxin A, and can also upregulate the expression of BIM and activate BAX, and TRIB3, leading to apoptosis. CHOP is transcriptionally activated by PERK via the phosphorylation of eIF2-α, which is also augmented by NH_4_Cl. Knockdown of CHOP or TRIB3 could also decrease VacA-induced mitochondrial dysfunction and apoptosis. CHOP is not only involved in apoptosis, but also in autophagy induced by VacA. Knockdown of the ER stress effectors, CHOP or TRIB3, could drastically decrease the formation of autolysosomes and cell death in VacA-treated gastric cancer cells. Therefore, VacA induces autophagy and cell death in the AGS cells by triggering ER stress, which involves the upregulation of CHOP and TRIB3 (119, 120).

Type I Shiga toxin produced by *Escherichia coli* can also cause apoptosis through endogenous and exogenous pathways, in which CHOP and DR5 play important roles. Silencing the expression of CHOP selectively blocks the activation of caspase and attenuates cell apoptosis (121, 132). Shiga toxins produced by *Shigella dysenteriae* serotype I activate both apoptotic cell death signaling and the ER stress response. Treatment of human retinal pigment epithelial cells (RPE cells) with Stxs results in the activation of JNK and p38MAPK, and up-regulation of CHOP and DR5 expression (123, 132). Collectively, characterization of CHOP functions during microbial infection will help us to understand the pathogenesis of microorganisms and provide a better theoretical basis to control and prevent diseases.

CHOP plays important functions during microbial infections, and therefore, may be an important potential target for new therapeutic approaches. Research has shown that the regulation of CHOP expression plays an important role in metabolic diseases and in some cancers (83, 133). CHOP deficiencies attenuate oxidative stress and renal ischemia-reperfusion-induced acute renal injury, *in vitro* and *in vivo* (83). The regulation of CHOP expression has been accepted as an approach to remove cancer cells through the induction of apoptosis (134). As mentioned above, small molecule inhibitors that inhibit ER stress (UPR) and the expression of CHOP may act as therapeutic options to prevent ER stress and microbial infections. Using the small molecule inhibitors, GSK2606414 or salubrinal, to inhibit the PERK-eIF2α pathway and the expression of CHOP can limit PCV2 replication (107). Thus, targeting CHOP may be a good therapeutic approach for the treatment of PCV2 infection. The chemical chaperone TUDCA is a classic ER stress inhibitor that improves ER folding capacity, which has been protective in various diseases, including diabetes mellitus, hypertension, calcification, and even cardiac dysfunction by preventing ER stress (135–137). TUDCA administration markedly suppresses cardiac ER stress and CHOP induction; thus, preventing cardiomyocyte apoptosis, cardiac inflammation and injury, cardiac dysfunction, reducing CVB3 replication *in vivo*, and increasing survival rates in CVB3 inoculation-induced AVMC models (113). Therefore, small molecule inhibitors that prevent ER stress and CHOP expression are potential therapeutic approaches for CVB3 infections. Additionally, in CHOP-deficient cells, the apoptosis caused by IBV was attenuated and IBV replication was limited and CVB3 replication was also limited (110, 113). Thus, overexpression or knock-out of the CHOP gene may be a therapeutic approach to treat related diseases. Therefore, understanding how CHOP functions during microbial infections will provide better therapeutic approaches to control and prevent diseases.

## Conclusions and Future Perspectives

**Figure 4:**
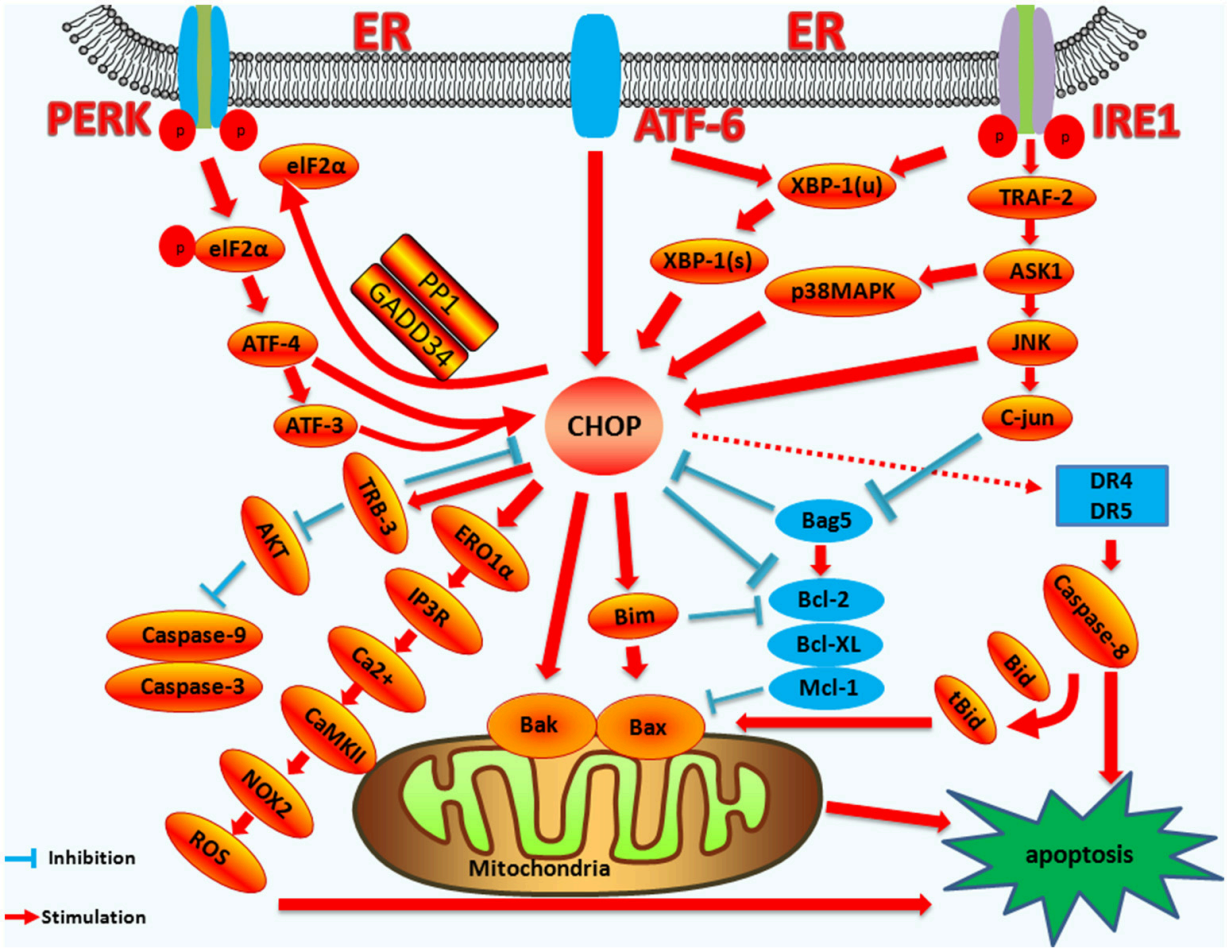
The mechanism of CHOP regulation in ER stress induced apoptosis. Upstream regulatory pathway: Activated PERK phosphorylates eIF2α, which results in the translation of ATF4. ATF4 translocates to the nucleus, which increases the expression of CHOP and ATF3. CHOP and ATF3 then bind to the promoters of the target genes, upregulating their expression. ATF6 translocates to Golgi apparatus where it is activated by proteolysis. Activated ATF6 transcriptionally upregulates CHOP expression. Additionally, ATF6 can regulate XBP-1 to activate CHOP. Activation of IRE1α processes unspliced XBP-1 mRNA into activated XBP1(s), which enters the nucleus and controls the expression of CHOP. IRE1α activates the apoptotic signaling kinase 1 (ASK1), which in turn phosphorylates p38 MAPK/JNK, and activates CHOP. In addition, Bag5 can reduce CHOP expression and increase Bcl-2 gene expression. Downstream regulatory pathway: CHOP triggers the intrinsic apoptotic pathway through the inhibition of BCL-2, BCL-XL, MCL-1, and the upregulation of BIM, which regulates BAX-BAK-mediated mitochondrial outer membrane permeabilization. This leads to cytochrome c release and the caspase cascade. CHOP can also regulate apoptosis by upregulating the expression of the TRB3 gene and preventing Akt phosphorylation, which inhibits the activity of caspases-3/9. CHOP triggers the extrinsic apoptotic pathway through the upregulation of DR4 and DR5, which regulate the caspase-8-mediated cascade. This leads to Bid cleavage into tBid, which regulates the BAX-BAK-mediated mitochondrial apoptosis pathways. CHOP can also trigger the ERO1α-IP3R-Ca^2+^-CaMKII pathway. ROS can also trigger Ca^2+^-dependent mitochondrial apoptosis. CHOP can directly activate GADD34 (DNA damage protein), which, combined with phosphatase 1 protein (PP1), dephosphorylates eIF2α, and results in protein translation recovery, increased ER stress, and cell apoptosis.

ER stress induced by pathogenic microorganism infection and subsequent apoptosis play pivotal roles in the regulation of infection (106, 138). CHOP is an important molecule in the ER stress-induced apoptosis pathway. CHOP-induced apoptosis also plays a pivotal role during virus or bacterium infection. Therefore, it is necessity to illustrate CHOP induced apoptosis pathway clearly. As mentioned above, there are extensive in-depth studies on the upstream regulatory genes of CHOP and its downstream target genes in the context of ER stress (a diagram summarizing the regulation of CHOP is shown in Figure 4).

Increasing evidence has suggested that infections are relevant to the abnormal expression of CHOP during ER stress and most of the time, the expression of CHOP can induce apoptosis. However, the mechanisms and pathways triggered by different pathogens vary and current researches indicate that CHOP plays an important role in apoptosis induced by pathogenic microorganisms. Some of those roles are beneficial to the microorganisms, while others are detrimental to the microorganisms. Most researches aim to show the apoptosis pathways induced by CHOP, and the functions of apoptosis to microorganisms. However, it is unknown under which conditions apoptosis induced by CHOP is beneficial to the release and spread of pathogenic microorganisms, or if such processes trigger a more intense immune response. Thus, how microorganisms use CHOP-induced apoptosis to regulate growth and reproduction, or how the host cells use CHOP-induced apoptosis to restrict a microorganism's replication and transmission are important aspects that need to be investigated further. The intricate balance between the two effects is worth further study, and the detailing of such mechanisms may help in the development of therapeutic approaches for the ER stress and microbial infection.

## Author Contributions

HH, MT, and CD drafted the review. SY critically revised the review. All authors read and approved the final manuscript.

### Conflict of Interest Statement

The authors declare that the research was conducted in the absence of any commercial or financial relationships that could be construed as a potential conflict of interest.

## Abbreviations

CHOP: C/EBP-homologous protein
ER: endoplasmic reticulum
ATF4: activating transcription factor 4
ATF6: activating transcription factor 6
DISC: death-inducing signaling complex
DR: death receptor
eIF2α: eukaryotic translation initiation factor 2α
IRE1: inositol-requiring enzyme 1
JNK: c-Jun N-terminal kinase 1
PERK: protein kinase RNA-like endoplasmic reticulum kinase
ROS: reactive oxygen species
TRAIL: TNF-related apoptosis-inducing ligand
UPR: unfolded protein response
XBP1: X-box-binding protein 1
XIAP: X-linked inhibitor of apoptosis protein
TRIB3: pseudokinase tribbles homolog 3
p38MAPK: p38 mitogen-activated protein kinase
CaMKII: calcium-dependent protein kinase
PDI: protein disulfide isomerase
FADD: Fas-associated death domain protein.
